# Genetics and epigenetics of *Pinus nigra* populations with differential exposure to air pollution

**DOI:** 10.3389/fpls.2023.1139331

**Published:** 2023-04-06

**Authors:** Elissavet Ch. Katsidi, Evangelia V. Avramidou, Ioannis Ganopoulos, Evangelos Barbas, Andreas Doulis, Athanasios Triantafyllou, Filippos A. Aravanopoulos

**Affiliations:** ^1^ Laboratory of Forest Genetics & Tree Breeding, Faculty of Agriculture, Forestry & Environmental Science, Aristotle University of Thessaloniki, Thessaloniki, Greece; ^2^ Laboratory of Plant Biotechnology – Genomic Resources, Hellenic Agricultural Organization DEMETER, Institute of Viticulture, Floriculture and Vegetable Crops, Heraklion, Greece; ^3^ Laboratory of Atmospheric Pollution and Environmental Physics (LALEP), Faculty of Engineering, University of Western Macedonia, Kozani, Greece

**Keywords:** *Pinus nigra*, AFLP, MSAP, air pollution, reproductive traits

## Abstract

Forest species in the course of their evolution have experienced several environmental challenges, which since historic times include anthropogenic pollution. The effects of pollution on the genetic and epigenetic diversity in black pine (*Pinus nigra*) forests were investigated in the Amyntaio – Ptolemais – Kozani Basin, which has been for decades the largest lignite mining and burning center of Greece, with a total installed generating capacity of about 4.5 GW, operating for more than 70 years and resulting in large amounts of primary air pollutant emissions, mainly SO_2_, NOx and PM10. *P. nigra*, a biomarker for air pollution and a keystone species of affected natural ecosystems, was examined in terms of phenology (cone and seed parameters), genetics (283 AFLP loci) and epigenetics (606 MSAP epiloci), using two populations (exposed to pollution and control) of the current (mature trees) and future (embryos) stand. It was found that cone, seed, as well as genetic diversity parameters, did not show statistically significant differences between the exposed population and the control. Nevertheless, statistically significant differences were detected at the population epigenetic level. Moreover, there was a further differentiation regarding the intergenerational comparison: while the epigenetic diversity does not substantially change in the two generations assessed in the control population, epigenetic diversity is significantly higher in the embryo population compared to the parental stand in the exposed population. This study sheds a light to genome dynamics in a forest tree population exposed to long term atmospheric pollution burden and stresses the importance of assessing both genetics and epigenetics in biomonitoring applications.

## 1 Introduction

Long-living forest species face during evolution several environmental challenges, some gradual and other abrupt. In recent times, the scientific community has focused on the humanly induced environmental changes that strongly affect forest species, especially keystone species that are the main determinants of sensitive natural ecosystems. Indeed, the health of natural forest tree populations are threatened not only by biotic (insects and diseases), but by abiotic (global climate change and pollution) factors as well (Aravanopoulos, 1998; Neale, 2007). Air pollution in particular, is as old as human civilization. Coniferous trees are the usual choice for biomonitoring and indicator species among higher plants (Smith, 1972; Rademacher, 2003; Piczak et al., 2003; Rodriguez Martin et al., 2018), as they retain their needles for two to five years, which is a fairly long period of exposure to pollution (Sawidis et al., 2001). Pine needles act as bio-accumulators and are therefore suitable as sensitive biomarkers to air pollution (Mingorance et al., 2007), and especially, to air pollution (Mingorance et al., 2007; Sun et al., 2009; Sun et al., 2010; Kuang et al., 2011).

Air pollution is quite significant in the region of Western Macedonia and in particular in the Amyntaio-Ptolemaida-Kozani basin (APKB), where the largest lignite deposits of Greece is concentrated. Close to 50-70% of Greece’s total electricity output has been produced in this region since 1950 (Triantafyllou, 2003; Nanos et al., 2015). The emissions arising from lignite burning and mining activities, cause pollution problems at the local level, while under specific atmospheric conditions prevailing pollutants are transported in longer distances outside APKB (Stalikas et al., 1997; Triantafyllou, 2003). The Greek government has recently decided the permanent retirement of lignite power plants, given the EU decarbonization policy implementation (Boulogiorgou et al., 2021); however the recent rally in natural gas and oil prices has already produced changes in the time frame of this policy. The area has suffered from high levels of airborne particles and heavy metals for about five decades (Stalikas et al., 1997), until power plants have been equipped with electrostatic precipitators of high retention efficiency. Black pine (*Pinus nigra* J.F. Arnold) forests are the most prevalent in the area and were found to present a higher capacity of absorbing and accumulating heavy metals (Cr, Mn, Fe, Ni, Cu, Cd and Pb) than other tree species in the APKB (Tsikritzis et al., 2002).

Field measurements of air pollutant concentrations are used to identify and quantify the impact of air pollutants on human health, agriculture and natural ecosystems. However, for a large-scale comprehensive assessment, modelling of pollutant transport is needed. Relevant models use mathematical and numerical techniques to simulate the physical and chemical processes that affect air pollutants as they disperse and react in the atmosphere. They are useful for investigating air pollutant transport, dispersion, deposition and finally, their temporal and spatial distribution (Daly and Zannetti, 2007; Oliveri, 2017). The advantage of these models is that they can also estimate air pollutant concentration and deposition in places where air quality is not measured for different reasons (cost, accessibility, etc.).

Air pollution exerts significant effects on the biome. While there is clear evidence that morphological features are very sensitive to selective pressures from the local environment (Aguinable et al., 1997), the clearest signal of environmental stress effects on natural populations comes from population genetics studies (Levene, 1953; Linhart and Grant, 1996; Feder et al., 1997; Fischer et al., 2013; Roda et al., 2013). Pollution affects the evolutionary process and can lead to changes in the genetic constitution of forest tree populations (Scholz et al., 1989; Aravanopoulos, 1998) especially with regards to the manifestation of natural selection (Scholz et al., 1989; Scholz and Bergann, 1984).

Rapid adaptation to environmental stress is also facilitated by epigenetic changes, which form a crucial biological mechanism that interconnects the environment, the genotype and the phenotype. Insofar, DNA methylation has been the most-studied epigenetic mechanism in ecology and population epigenetics (Kilvitis et al., 2014; Avramidou et al., 2015a). There is a strong indication that methylation changes in plants exposed to heavy metal pollutants (Labra et al., 2002), while variance in DNA methylation has also been increased in response to ecologically relevant stressors (Vandenhove et al., 2010; Dowen et al., 2012; Herrera et al., 2013; Xie et al., 2015). Moreover, several studies indicate that epigenetic diversity caused by environmental stress can be inherited to offspring or future generations (Boyko and Kovalchuk, 2008; Gutzat and Mittelsten Scheid, 2012), even in the absence of the original stress conditions (Hauben et al., 2009; Vandenhove et al., 2010; Vanyushin and Ashapkin, 2011; Bilichak et al., 2012).

Herein, we selected the Amyntaio-Ptolemaida-Kozani Basin as our study area, due to its high levels of air pollution for a prolonged period of time. We used a prognostic air pollution model (TAPM) for investigating the footprint of SO_2_, NO_x_ and PM10 (particulate matter). We selected *P. nigra* as a biomarker in order to assess quantitative morphological parameters, as well as population genetic and epigenetic diversity using molecular markers, and evaluate possible changes in the black pine forest stands triggered by accumulated pollution stress lasting for the past 70 years. Furthermore, we also studied the epigenetic profile of the next generation (embryos), to investigate if DNA methylation changes were transmitted and/or persisted.

## 2 Materials and methods

### 2.1 Population selection and sampling

The air pollution model TAPM (Hurley et al., 2001) was used for investigating the footprint of SO_2_, NOx and PM10 on the *P. nigra* natural populations in the APKB. In total, eight locations that correspond to the natural distribution of *P. nigra* in the APKB area were investigated (**Table 1**). TAPM is an operational, three-dimensional (3D) terrain following sigma-coordinate, coupled prognostic meteorological and pollutant dispersion model, that uses the fundamental equations of atmospheric flow, thermodynamics, moisture conservation, turbulence, and dispersion (Hurley et al., 2001; Hurley et al., 2003a; Hurley et al., 2003b). The model was configured, ran and validated in the area of interest by Triantafyllou et al. (2011); Aidoui et al. (2015) and Matthaios et al. (2017). Based on the model results tailored to this study, out of the eight locations studied, the two areas - receptors of natural *P. nig*ra populations, associated with the extreme concentrations (maximum and minimum), were selected, following a methodology adapted from Rajput and Agrawal (2005).

**Table 1:** Average annual concentrations predicted by the TAMP model, for PM10, SO2 and NOx, for eight sites that host natural *Pinus nigra* populations in the Amyntaio-Ptolemaida-Kozani basin (APKB), Western Macedonia, Greece.

Average annual concentrations	Mpourika	Vourinos	Flampouro	Vlasti	Titaros 1	Titaros 2	Agia Kyriaki	Palaiogratsano
**PM10 (μg/m3}**	11.083	23.174	9.050	8.5	9.327	8.317	8.240	7.731
**SO_2_ (ppb)**	1.623	1.223	1.035	1.57	0.359	0.773	0.384	0.433
**NOx (ppb)**	0.177	0.075	0.116	0.069	0.027	0.035	0.329	0.026

### 2.2 Sample collection - trees

Sampled trees from APKB were chosen to be at least 30 m apart in order to avoid sampling from related individuals, while isolated trees (no other tree in a radius of 25 m) were excluded from sampling. All sampled individuals were mature trees (average tree age: 80 years) of the dominant class having similar DBH and tree height. A total of 24 trees were sampled per population in terms of needles (10 per tree) and cones (three per tree) for seed extraction.

For the genetic and epigenetic analyses, a total of N=48 samples per population were used. Each population sample comprised of 24 samples from needle and embryo tissue (the latter excised from seeds), respectively.

### 2.3 Morphological measurements

The following parameters were assessed: (1) number of scales, (2) percentage of full seeds (out of 1000 seeds) and (3) weight of 1000 seeds (taking into account filled seeds only).

### 2.4 Sample collection - soil

In total, 70 soil samples were collected per soil layer per population, 35 from the top 5 cm of soil and 35 corresponding to a 5 -10 cm depth. A total of 48 soil samples were collected at a 3 m distance from the trunk of each sampled tree while 11 additional samples per population were randomly collected from the two sites. The samples were oven dried at 85°C and then homogenized and pulverized using different diameter sieves. The concentrations of Ca, Mg, K and Na were implemented using sequential leaching and those of Cu, Mn, Zn, and Fe by the DTPA method (Lindsay and Norwell, 1978). The concentrations of these elements were determined by atomic absorption spectrophotometry. The pH was determined using the electrometric (potentiometric) method and a calcimeter was used to estimate carbonates.

### 2.5 DNA extraction

Total genomic DNA of each individual was extracted and isolated from needles and embryos using the Dellaporta protocol (Dellaporta et al., 1985). For a genome-wide surveying of genetic and epigenetic variation, f-AFLP and f-MSAP markers (Foust et al., 2016), were used.

### 2.6 Genetic analysis

AFLP analysis was performed according to Avramidou et al (2015a; 2021). A primer pair based on the sequences of the EcoRI and MseI adapters with one additional selective nucleotide at the 3´ end (EcoRI+A and MseI+C) was used for the first PCR step (pre-amplification) (**Table S1**). Cycling was performed on a Eppendorf thermocycler with a 94°C hold for 30 sec, followed by 23 cycles of 94°C for 30 sec, 56°C for 30 s and 72°C for 1 min and successively followed by a final hold at 72°C for 7 min. Selective amplifications were carried out in 10-μl total volumes consisting of 3 μl of diluted preselective template and using the same reaction conditions as for preselective amplification, but using 30 ng of an MseI primer and 5 ng of an EcoRI primer per reaction (**Table S2**). Selective amplification cycling was accomplished on a Eppendorf thermocycler with the following program: an initial cycle of 94°C for 30sec, 65°C for 30 sec, 72°C for 1 min; then twelve cycles of 94°C for 30 sec with an annealing temp starting at 65°C for 30 sec, but decreasing by 0,75°C each cycle, 72°C for 1 min; finally, 23 cycles of 94°C for 30 sec, 56°C for 30 sec, 72°C for 1 min, with a final hold at 72°C for 30 min.

### 2.7 Epigenetic analysis

The detection of methylation-sensitive amplified polymorphisms was based on Avramidou et al. (2015b; 2021). For the f-MSAP assay the same needle DNA samples were used and the whole procedure (as described above for AFLP) was repeated for the two different combinations of EcoRI/HpaII and EcoRI/MspI. The different adapters and the four sequences of primers that have been used for preselective and selective amplifications are presented in **Tables S3** , **S4**.

### 2.8 Data analysis

Descriptive statistics of soil related variables, as well as of cone and seed parameters, were calculated. Population differences were assessed using *t*-tests for independent samples, while percentages were evaluated using two sample *t*-test between percentages. The StatPac software (http://www.statpac.com) was employed.

Presence/absence of alleles of f-AFLP product mixtures was scored as “1/0”. Only reproducible fragments ranging from 150 to 500 bases were counted (Vekemans et al., 2002). Monomorphic fragments were excluded from further analysis (Bonin et al., 2004). Genetic parameters were estimated using the GenAlEx 6.5 software (Peakall and Smouse, 2012). The genetic differentiation between populations was determined by AMOVA using Φ_PT_ with 9,999 permutations, a measure suitable when comparing different data types (Teixeira et al., 2014), in this case genetic and epigenetic data.

F-MSAP products were scored in a similar manner. To separate unmethylated and methylated fragments and to test for the impact of different methylation conditions, we used the ‘Mixed-Scoring 2’ approach (Schulz et al., 2013). Epigenetic diversity within populations was quantified using the MSAP_calc scenario of the R programming language (Schulz et al., 2013) as follows: (i) number of total and private bands (polymorphic subepiloci), (ii) percentage of polymorphic subepiloci (P_epi_) and (iii) mean Shannon’s information index (I_epi_). GenAlEx 6 (Peakall and Smouse, 2012) was employed to compute haploid gene diversity (h) within and between populations. GenAlEx was also used to conduct an analysis of molecular variance (AMOVA) - separately for each subepilocus class - in order to study the variation of CCGG methylation states (epiloci) between populations. Separate principal coordinate analyses (PCoA) were employed to assess differentiation among populations and among generations (needles and embryos), as well as on the different subepilocus classes, following Ganopoulos et al. (2011). The percentages of epigenetic variability between and within the populations were estimated with the differentiation coefficient Φ_PT_.

## 3 Results

### 3.1 Application of the TAPM model

The spatial distribution of concentrations following the application of the dispersion and pollution model (TAPM), was evaluated to locate the population most exposed to air pollution and the one with minimum exposure (control) (Rajput and Agrawal, 2005).

**Figure 1:**
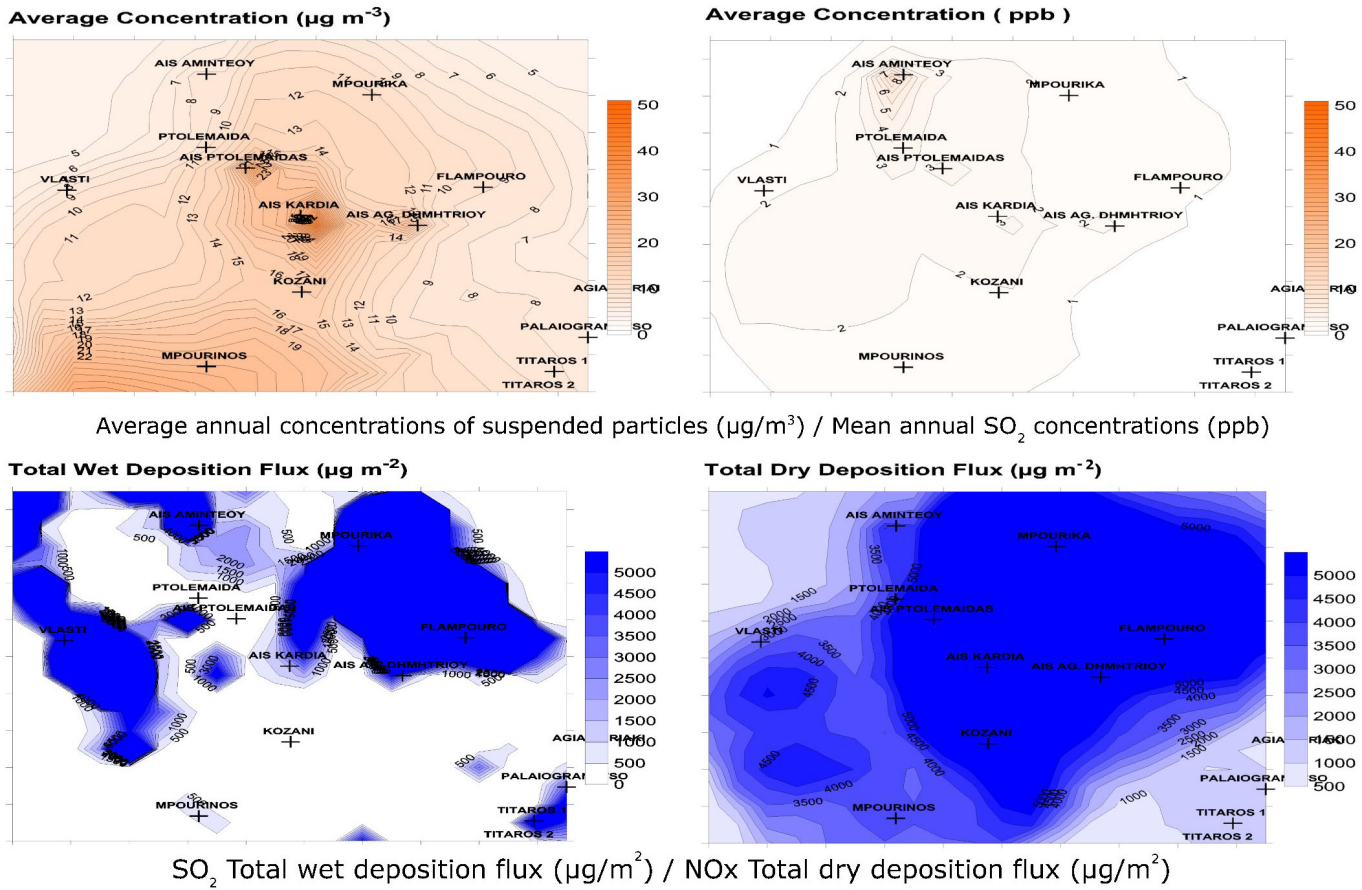
Mean annual PM10 concentrations, Mean annual SO2 concentrations, Total SO2 wet deposition flux, Total NOx dry deposition flux, based on the prognostic air pollution model (TAPM) in the in the Amyntaio-Ptolemaida-Kozani basin (APKB), region of Western Macedonia, Greece.

The mean annual concentrations of PM10 (particles with aerodynamic diameter less than 10 microns), the mean annual concentrations of SO2, the dry deposition of NOx and the wet deposition of SO2, as predicted by the TAMP model, are presented in **Table 1**; **Figure 1**.

The population of Mpourika was chosen as the one with the maximum exposure to pollution and the population of Agia Kyriaki as the control, among nine candidate natural populations of *P. nigra* in the APKB. Although the values as predicted by the model were generally not high, they differ notably in these areas, for instance the SO_2_ concentration is 4.2x higher in the former. The two areas present the same average altitude of 1500 m, and prevailing climatic conditions were similar (**Table S5**) Regarding soils Mpourika presented sedimentary rock and luvisols and Agia Kyriaki had gneiss and soils originating from crystalline rocks.

### 3.2 Soil analyses

The results of the soil analysis are presented in **Table S6**. The soil analysis performed on the two sites, showed that the concentrations of the elements are within normal limits and there is no visible effect of soil pollution.

### 3.3 Cone and seed parameters

The results of the quantitative morphological parameters analysis are presented in **Table 2**. The differences in the numbers of fertile cone scales, the numbers of full seeds and the weight values of 100 seeds, between the exposed to pollution population (Mpourika) and the control (Agia Kyriaki) were not statistically significant.

**Table 2:** Descriptive statistics of cone and seed parameters of the selected *Pinus nigra* populations of maximum exposure (Mpourika) and control (Ag. Kyriaki) in the APKB.

Quantitative morphological parameters	Population	N	Mean	SD	SEM	T-test value/p
Number of fertile cone scales	Mpourika	49	80.54	10.66	1.52	1.602/0.613
	Agia Kyriaki	39	84.32	11.40	1.82	
Percentage of full seeds	Mpourika	49	50.76	27.87	3.98	0.453/0.652
	Agia Kyriaki	39	45.9	21.27	3.40	
Weight per 1.000 seeds	Mpourika	49	21.6	12.6	1.8	0.412/0.681
	Agia Kyriaki	39	20.74	3.67	0.58	

### 3.4 Population genetic analysis

**Figure 2:**
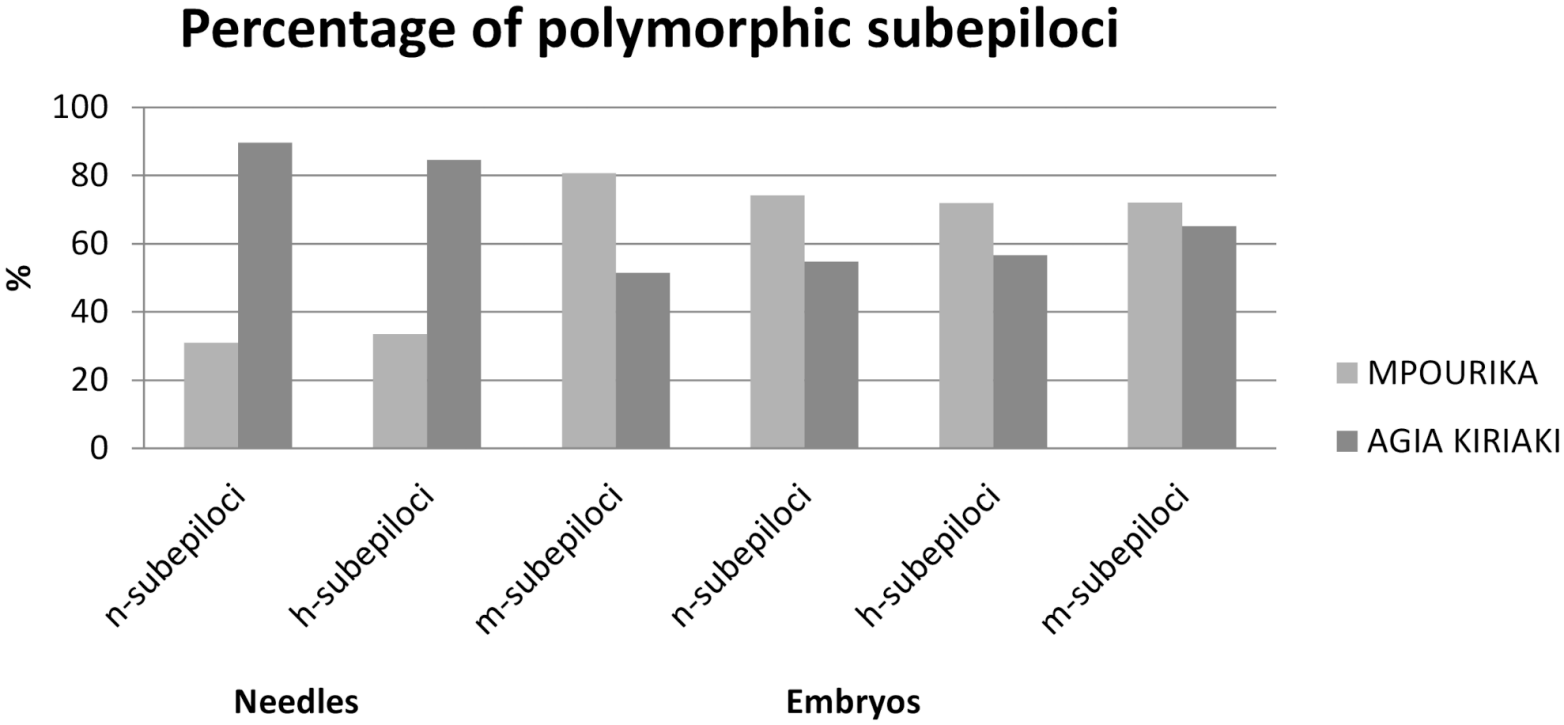
Percentage of polymorphic n-, h- and m-subepiloci in the exposed (Mpourika) and control (Agia Kyriaki) populations for the mature (needle tissue) and future (embryo tissue) stands.

The AFLP analysis revealed a total number of 283 amplified fragments, out of which 208 (73.5%) showed polymorphism in the exposed population and 212 (74.9%) were polymorphic in the control population. The slight decrease in the percentage of population polymorphism that was found in the exposed population was insignificant. The values of the basic population genetic parameters are reported in **Table 3**. The exposed population presented somewhat lower values in the genetic diversity parameters studied, but there were no statistically significant differences between this population and the control (**Table 3**). Almost all variation resided within populations (Φ_PT_=0.001; **Table S7**). Gene flow between the two populations was very high (Nm estimate based on Φ_PT_, is Nm=249.75) and the two populations appear to have practically zero genetic distance. The orientation of the populations is presented by a PCoA in **Figure 2**.

**Table 3:** Genetic and epigenetic diversity parameters of the selected *Pinus nigra* populations in the APKB (N, sample size; P, percent polymorphic loci; Na, number of alleles; Ne, effective number of alleles; I, Shannon diversity index; He, Expected Heterozygosity; h_epi_, epigene diversity; standard error in parentheses).

	N	AFLP (needle tissue)							MSAP (needle tissue)					MSAP (embryo tissue)				
Population		No. bands	No. private bands	P	Na	Ne	I	He	No. bands	No. private bands	P_epi_	I_epi_	h_epi_	No. bands	No. private bands	P_epi_	I_epi_	h_epi_
Mpourika	24	209	19	73.50	1.470	1.074	0.123	0.062	346	193	57.10	0.090	0.043	478	345	72.18	0.118	0.058
					(0.053)	(0.006)	(0.008)	(0.004)				(0.004)	(0.002)				0.005	(0.003)
Agia Kyriaki	24	214	23	74.91	1.498	1.082	0.134	0.068	413	260	68.15	0.107	0.051	478	291	60.88	0.102	0.058
					(0.052)	(0.006)	(0.008)	(0.005)				(0.004)	(0.002)				0.005	(0.003)
Mean	24	211.5	21	74.20	1.484	1.078	0.129	0.065	379.5	226.5	62.63	0.099	0.049	478	318	66.53	0.110	
							(0.03)											
t-test (population comparison)					0.041	0.073	0.061	0.065				2.999	0.071				2.259	0.077
p					0.486	0.474	0.478	0.477				0.004	0.940				0.029	0.945

T-tests statistic and associated p-values for population comparisons are indicated at the bottom of the Table.

### 3.5 Population epigenetic analysis - 1: Needles

**Figure 3:**
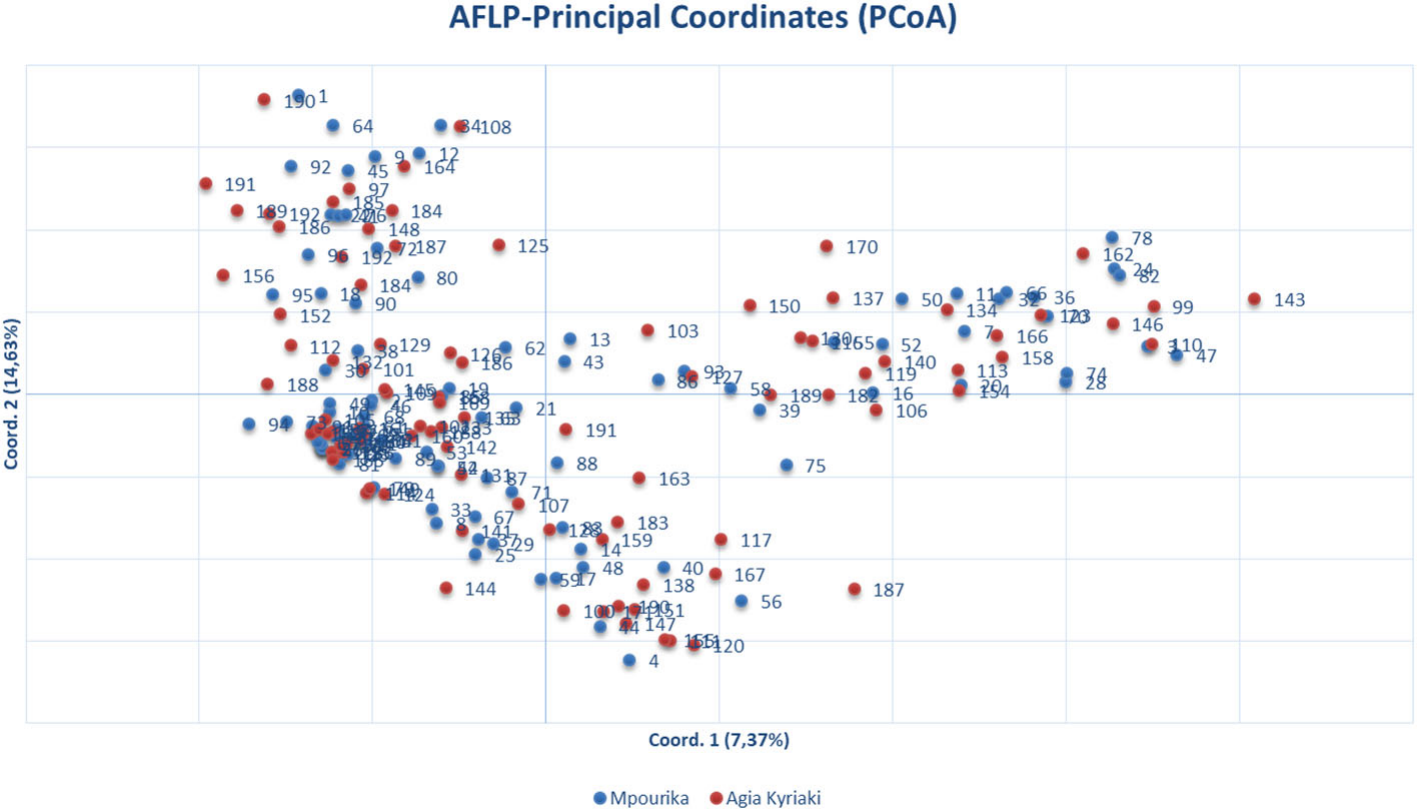
Principal coordinate analysis (PcoA) of genetic distances based on AFLP analysis of needle tissue in the exposed (Mpourika) and the control (Ag. Kyriaki) populations.

The epigenetic diversity statistics are also presented in **Table 3**. MSAP analysis using needles, revealed 606 stable and repeatable epiloci. The exposed population revealed 346 polymorphic epiloci (P_epi_=57.1%) and the control population 413 (P_epi_=68.15%). Total methylation polymorphisms that pertain to semimethylated (h) and fully methylated (m) epiloci amounted to P_epi_=57.06% for the exposed population and P_epi_=68.02% for the control populations, respectively (**Figure 3**). It is noted that while n-epiloci polymorphism was higher in the control population, m-epiloci polymorphism was higher in the exposed population. The epigenetic Shannon index (I_epi_) showed statistically significant differences in diversity between the exposed and the control populations when assessing the mature stand, as shown by a t-test (**Table S8**).

**Figure 4:**
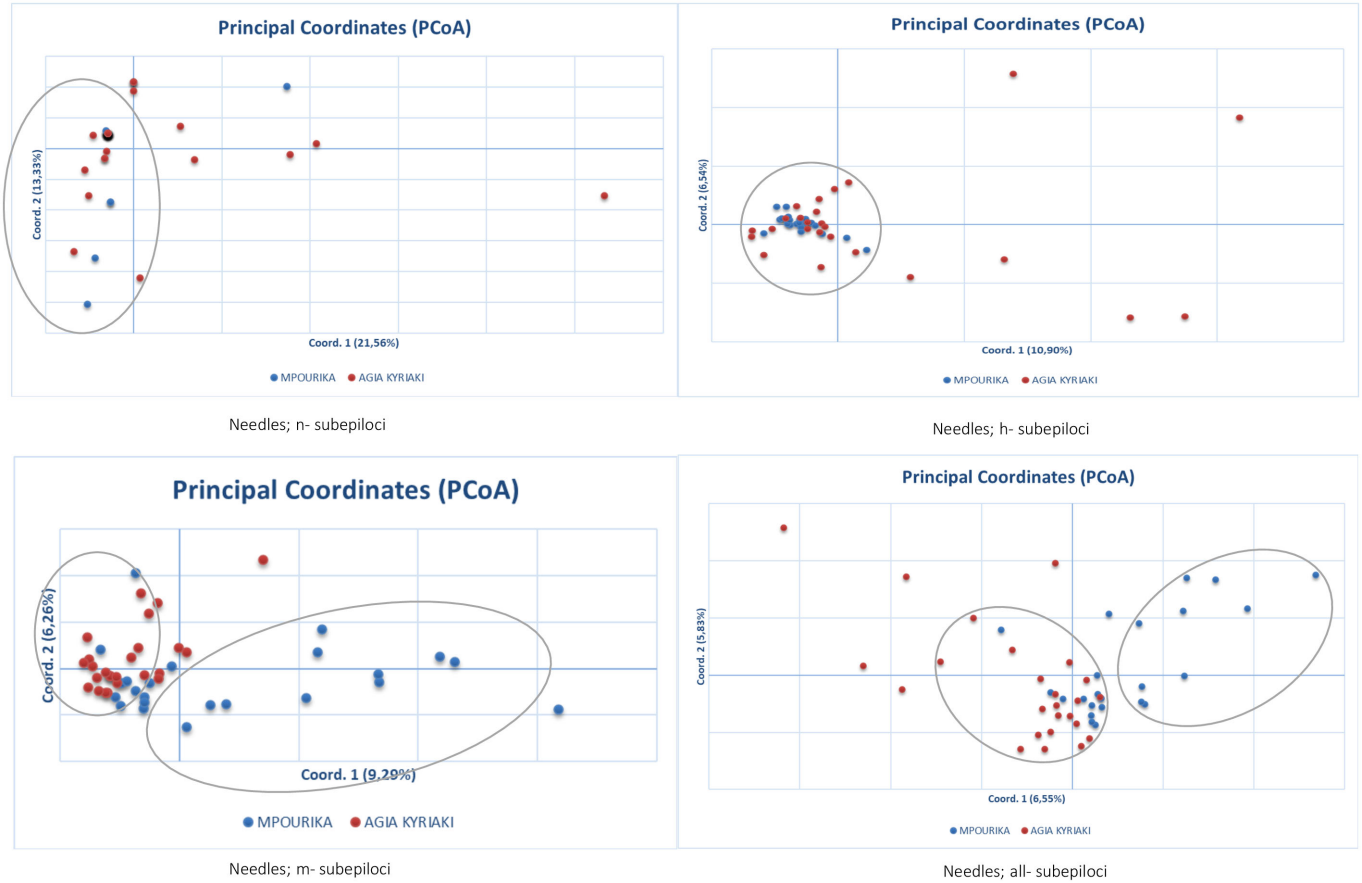
Principal coordinate analysis (PcoA) of epigenetic distances based on MSAP analysis, of needle tissue, in the exposed (Mpourika) and the control (Ag. Kyriaki) populations.

The epigenetic differentiation value between the two populations was low, but significant (Φ_PT_=0.034, p=0.001). Differentiation was also studied in the subepiloci level. AMOVA and Φ_PT_ results for n-subepiloci, h-subepiloci and m-subepiloci (Φ_PT_=0.034, p=0.001), showed that the differentiation between the exposed and control mature populations was small as well (**Table S7**). The differentiation of the two populations in multivariate space was studied by PCoA for the n-, h- and m- subepiloci, as well as for all sub-epiloci (**Figure 4**). The explanation of total variation was rather low (34.8%, 17.4%, 15.5% and 12.39%, respectively). Although no clear population distinction was seen, the ordinations at two-dimensional space differ. The PCoAs of the n- and h- subepiloci (non-methylated and hemi-methylated respectively), show no particular groupings and the presence of a large conglomerate group consisting of individuals from both populations. On the other hand, the PCoAs of the m-subepiloci (methylated) and all subepiloci depict two loosely defined groups: one consisting of individuals from both populations, and another that includes individuals from the exposed population.

### 3.6 Population epigenetic analysis - 2: Embryos

The analysis revealed 478 stable and repeatable epiloci. The exposed population showed 345 polymorphic epiloci (P_epi_=72.18%) and the control population 291 (P_epi_ = 60.88%). Total methylation polymorphisms, i.e., semi methylated (h) and fully methylated (m) epiloci polymorphism amounted to P=72.02% for the exposed population and P=60.9% for the control population (**Figure 3**). In all epiloci polymorphism was higher in the exposed population in embryos. This result is in contrast to the mature populations where only polymorphism in m-subepiloci was higher in the exposed population. The epigenetic Shannon index (I_epi_) showed statistically significant differences in diversity between the exposed and the control embryo populations as well (shown by a t-test; **Table S8**).

**Figure 5:**
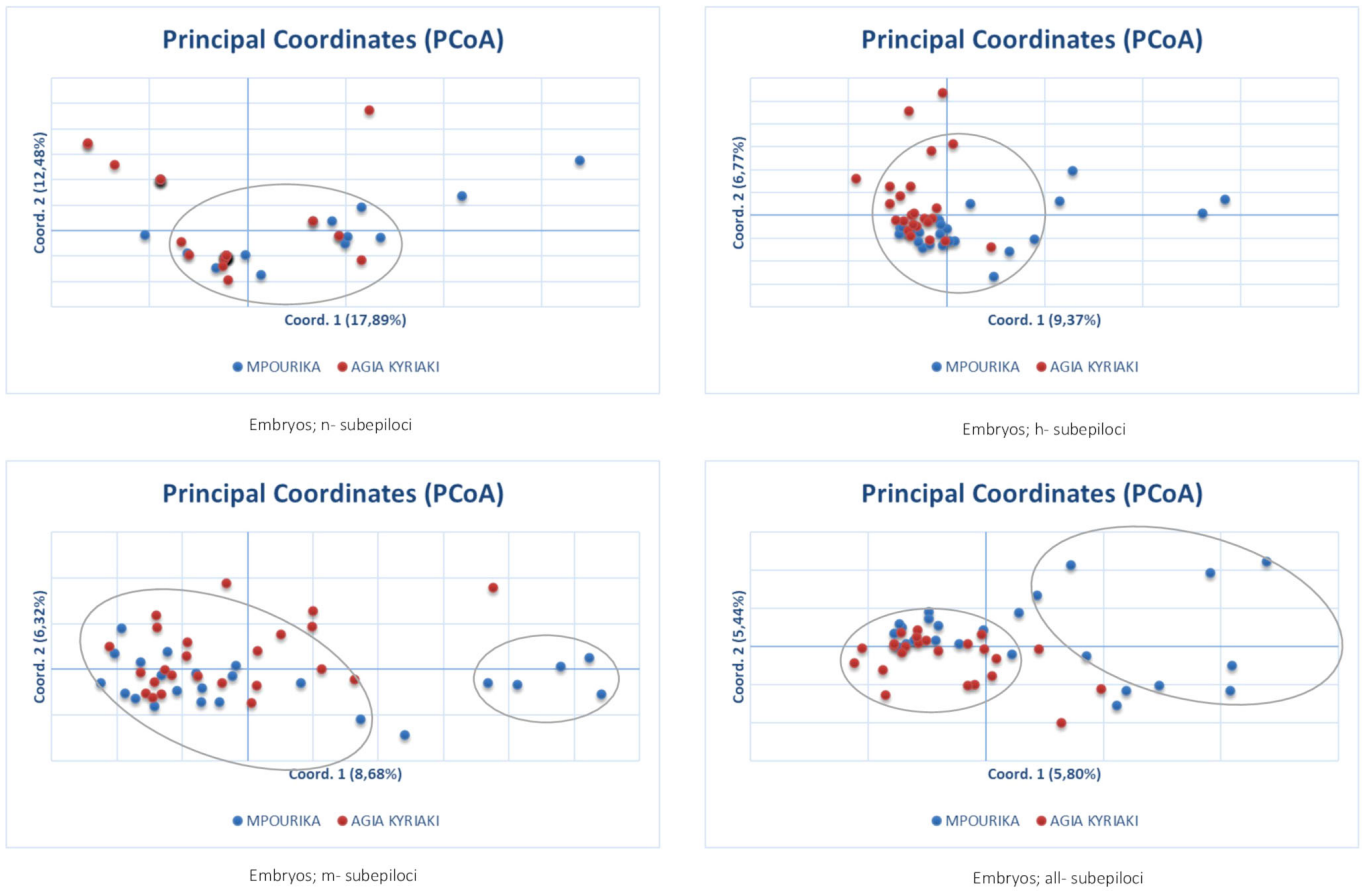
Principal coordinate analysis (PcoA) of epigenetic distances based on MSAP analyses of embryo tissue, in the exposed (Mpourika) and the control (Ag. Kyriaki) populations.

The epigenetic differentiation value was Φ_PT_=0.021 (p=0.002) for embryo tissue, which implies weak, but significant differentiation. AMOVA results and Φ_PT_ for n-subepiloci, h- subepiloci and m- subepiloci (Φ_PT_=0.021, p=0.002), showed that there was some differentiation between the two embryo populations (**Table S7**). The differentiation of the two populations’ embryos in multivariate space was studied by PCoA analyses of n-, h-, m- and all subepiloci (**Figure 5**). The explanation of the total variation was also low (30.4%, 16.1%, 15.1% and 11.23% for n-, h-, m- and all subepiloci respectively). As seen in the respective analyses of mature trees (needle tissue) above, the PCoAs of the n- and h- subepiloci (non-methylated and hemi-methylated respectively), show no particular groupings and the presence of a large conglomerate group of embryos from both populations. As above, loosely defined groups could be identified in the PCoAs of the m-subepiloci (methylated) and all subepiloci, one group consisting of embryos from both populations and the other of individuals principally from the exposed population. I

## 4 Discussion

### 4.1 Soil analyses

The AKPB area has been studied extensively (Georgakopoulos et al., 2002a; Georgakopoulos et al., 2002b; Triantafyllou, 2003; Samara, 2005), as the area was one of most affected in Greece by gaseous pollutant emissions. In the past, the area presented high levels of airborne pollutants and heavy metals (Tassiou et al., 1985; Stalikas et al., 1997). Heavy metals emitted in α gaseous form associated with fly ash, can be transported in long distances before deposition. Nevertheless, soil impacts are more likely to be higher in the source area (Swaine, 1994; Mandal and Sengupta, 2006). The average distance of deposition from the emission source is related to many parameters, including the characteristics of a particular source (e.g. chimney height), meteorological conditions and topography (Godin et al., 1985).

Overall, the observed concentrations of gaseous pollutants in the studied populations do not affect the concentrations of the key soil components. There is some significant variation in pH and in concentrations of certain elements (Ca, K, Mn and Cu), but this is likely the result of the geological background of each region, a finding consistent with pertinent literature (Rodríguez Martín et al., 2007; Petrotou et al., 2012; Nanos et al., 2015).

### 4.2 Cone and seed parameters

The number of fertile cone scales is an indication of a population’s fecundity and indirectly of the (absence of) inbreeding (Mosseler et al., 2000; Major et al., 2005; Lee et al., 2018). The percentage of filled seeds and the weight per 1000 seeds, also provide an indication of population status, as statistical differences among populations may indicate differential selection pressure and may also pinpoint towards differences in effective population size (Aravanopoulos et al., 2020). The weight per 1000 seeds range, is within the values reported for black pine populations of the Mediterranean basin (Aguinable et al., 1997; Turna et al., 2006). Aguinable and Bueno (1994) pointed out that the weight of 1000 seeds successfully separated different populations and that such morphological features are very sensitive to selective pressures from the local environment. Herein, the differences in the three cone and seed parameters studied between the exposed to pollution and control populations were not statistically significant. Given that these populations grow at the same elevation and under similar climatic conditions, the differential effect of the atmospheric pollution does not seem to have a direct influence on the studied parameters linked to reproductive capacity and population viability.

### 4.3 Population genetic analysis

The number of loci detected in this study was within the range reported for other studies that have used similar sets and numbers of primers, e.g. for *Pinus pinaster* (Costa et al., 2000), *Pinus brutia* (Choumane et al., 2004), *Pinus taeda* (Cervera et al., 2000), *Pinus echninata* (Xu et al., 2008), and *Pinus roxburghii* (Rawat et al., 2014). The polymorphism found is within the range reported in the literature for *P. nigra* regarding dominant markers (Nybom, 2004). Population polymorphism was somewhat lower in the exposed population, a finding consistent with the results of Kuchma and Finkeldey (2011) and Piraino et al. (2006), where the populations of maximum exposure were less polymorphic than the control population.

The values of population genetic parameters were generally lower than those observed in other studies. For instance, the values of the effective number of alleles were lower than those observed by Dias et al. (1.5896; 2019) and Rubio-Moraga et al. (1.305; 2012), in *P. nigra*, but for ISSR markers. Also, in the present study the expected heterozygosity (He) was lower than the values of Rubio-Moraga et al. (0.175; 2012). Furthermore, the Shannon diversity index (I) was also lower compared to previously published papers from Rubio-Moraga et al. (0.199 to 0.366; 2012) and Dias et al. (0.3458 to 0.5186, 2019). On the other hand, the number of different alleles (Na) was within the range of Rubio-Moraga et al. (1.219; 2012).

When comparing the two populations, the exposed population presented somewhat lower values in the genetic diversity parameters studied, but there were no statistically significant differences. The same finding was also reported in the study of Kuchma and Finkeldey (2011) who used AFLP markers to study populations differentially exposed to radiation pollution in *P. sylvestris*. Gene flow between populations was very high. Gene flow is generally very high in forest trees over long distances (Kremer et al., 2012), a finding also observed in *P. nigra* where strong recent and historical gene flow coupled with weak spatial structure was revealed (Scotti-Saintagne et al., 2019). The orientation of the populations by PCoA in two-dimensional multivariate space, showed that there is no apparent population differentiation. This result, in concordance to the finding that almost all variation resided within populations, can be regarded as a further indication of the absence of notable impact by pollution at the genetic level.

In populations that grow in similar altitude and climatic conditions and present strong gene flow, absence of significant differences in their neutral genetic diversity can be expected, except of course for genes under differential selection pressure (in this case, pollution stress). The *P. nigra* populations of APKB do not appear to have been notably affected in their genetic diversity and differentiation (as seen by neutral genetic markers), by pollution from lignite power stations and mines that has been ongoing for about the past 70 years. It is unclear if the detection of rather lower neutral diversity values in the exposed population can be considered as an early sign of a reaction to selective pressures from the gaseous pollutants in the region, in the presence of some genetic hitchhiking event.

### 4.4 Population epigenetic analysis - 1: Needles

Epigenetic diversity as shown by the Shannon index (I_epi_) significantly different between the exposed and the control populations. Total epigenetic differentiation between the exposed and control populations was low but significant, a result that was also found at the subepiloci level. While a higher percentage of the epi-variation resided within populations, this value was less than that found in the respective genetic analysis, where almost all variation resided within populations (**Table S7**). The differentiation of the two populations in multivariate space by PCoA showed that while for the non-methylated and hemi-methylated subepiloci there were particular groupings, in the case of the methylated subepiloci and all subepiloci two loosely defined groups (one including individuals from both populations and one including individuals from the exposed population) were depicted. This result indicates that full methylation is associated with some differences in the ordination in principal coordinate space between the exposed and control populations.

Low but significant differentiation was also found in different populations of *Prunus avium* (Avramidou et al., 2015). Significant epigenetic differences between natural populations have been reported in other studies, for example in *Populus alba* (Ma et al., 2013), Solanum sp. (Cara et al., 2013) and *Hordeum brevisubulatum* (Li et al., 2008). Chwedorzewska and Bednarek (2012) found a strong epigenetic differentiation (Φ_ST_​​=0.50) between populations of *Poa annua*, while Richards et al. (2012) reported a strong epigenetic differentiation (Φ_ST_=0.50-0.80) on natural populations of *Fallopia japonica* grown in different environments (Richards et al., 2012). Schulz et al. (2014) consider that the changes caused by environmental factors can lead to a reduction of epigenetic differentiation of populations that grow in similar habitat conditions, a view supported by insofar published literature and the results of this research.

### 4.5 Population epigenetic analysis - 2: Embryos

MSAP analysis on embryos was performed in order to assess the epigenetic status of the next generation. Epigenetic diversity (assessed by the Shannon index, I_epi_), was significantly different between the exposed and control populations. More importantly, while the epigenetic diversity does not substantially change in the two generations assessed in the control population (t=0.781, p=0.439), this is not the case in the exposed population. In the latter, epigenetic diversity increases considerably in the embryo population (t=4.373, p=0.001), showing a significant potential increase in the forthcoming generation.

The epigenetic differentiation value between the exposed and control stands for the embryo populations, was low but significant as observed in the mature populations as well. At the subepiloci level the differentiation was also significant but low, less than the differentiation observed in the partitioning of epi-variation in the mature stands (**Table S7**). The differentiation in multivariate space as studied by PCoA showed similar results to the respective analyses of mature trees (needle tissue). Loosely defined groups could be identified only in the PCoAs of the methylated subepiloci and all subepiloci, one including embryos from both populations and the other individuals principally from the exposed population. It appears that also in the embryos, full methylation is associated with some difference in the ordination in principal coordinate space between the exposed, and control populations.

### 4.6 Comparison of population genetic and epigenetic analyses

The comparison of genetic and epigenetic diversity (**Table 3**), showed that the average percent of polymorphism and the mean genetic diversity of the two populations was higher in the genetic analysis (P=74.20% vs. P_epi_=62.6% and h=0.065 vs. h_epi_=0.049, respectively). The results agree with Rico et al. (2014) who studied natural populations of *Quercus ilex* subject to drought and found a reduction of polymorphism in epiloci, but not in genetic loci. Epigenetic variation on a total population basis (including both the analysis of the mature and the future stand), was also assessed by the Shannon index. Epigenetic diversity was found to be numerically lower than genetic diversity (**Table S9**), but this difference was not statistically significant (t=0.369, p=0.713 for the exposed population, and t=0.337, p=0.737 for the control population). The presence of comparable genetic and epigenetic diversity according to the Shannon index, is in agreement with other similar studies (e.g. Herrera and Bazaga, 2010; Herrera et al., 2013; Schultz et al., 2013; Wenzel and Piertney, 2014; Avramidou et al., 2015).

### 4.7 General discussion and conclusions

The genetic analyses have shown that a small decrease was detected gene diversity of the exposed population compared to the control, although the observed differences are numerical and not-statistically significant. Neutral inter-population genetic diversity differences are generally not expected under the strong gene flow observed. Epigenetic analysis has depicted that methylation in the exposed population was higher both in needles and embryos compared to the control population, and the differences in the relevant epigenetic parameters analysed were statistically significant. These differences may also be reflected to some extend by the results of PCoA analyses, as the two populations are not differentiated at the genetic level, but some differentiation is seen when methylated epiloci are considered. The higher percentage of total methylation observed in embryos (and the presence of higher polymorphism in all subepiloci in the exposed population of embryos in contrast to the respective results in mature trees), may be an indicator of the next-generation adaptability to current local environmental conditions. In this respect, our study may reveal a case where the evolutionary dynamics are first driven by spontaneous epigenetic modifications which arise at faster rates than genetic changes (Klironomos et al., 2013; Richards et al., 2017). Our results are in agreement with Labra et al. (2004) who found hypermethylation on *Brassica napus* that was exposed to potassium dichromate in relation to the control. It seems that hypermethylation, or an increase in methylation, is triggered from environmental pressures and may be associated with adaptation. Moreover, Georgieva et al. (2017) found a 10% increase and a connection to adaptation when they evaluated CCGG methylation patterns between radio-contaminated Chernobyl and control plants of soybean.

Previous studies have suggested that methylation polymorphism in plant genomes is a confounding result of adaptation due to environmental stress (Finnegan et al., 1996; Kovarik et al., 1997; Labra et al., 2002; Wada et al., 2004; Chinnusamy and Zhu, 2009; Angers et al., 2010; Kooke et al., 2015). Diversity in DNA methylation directly links the genome to the environment and DNA methylation has relevance as a biomarker of past and present environmental stress (Rey et al., 2020). Epigenetic changes lead to changes in gene expression, which are sensitive to environmental change (Boyko and Kovalchuk, 2008), while DNA methylation is considered to be one of the main mechanisms responsible for conifer evolution (Nystedt et al., 2013). Genome methylation can also be a reversible process, influenced by complex gene - environmental interactions (Ramchandani et al., 1999). Overall, few attempts have been made to relate genetic and epigenetic variation in natural plant populations (Shan et al., 2011; Ma et al., 2013; Rico et al., 2014; Avramidou et al., 2015). Even fewer studies so far, have examined the coupling of genetic and epigenetic diversity in natural populations under the influence of environmental stress (Gao et al., 2010; Herrera and Bazaga, 2010; Lira-Medeiros et al., 2010). Such studies are crucial in order to clarify the relationship between genotype, epigenotype and phenotype for forest species and other plants, especially under conditions of stress.

In view of the upcoming climate change, epigenetic processes can provide a quick response machinery, allowing evolutionary mechanisms to lead to rapid micro-evolution and adaptation of populations (Koch et al., 2014). Accumulating evidence suggests that DNA methylation can affect ecologically important traits, such as stress tolerance, even in the absence of genetic variation (Kilvitis et al., 2014), and that DNA methylation can contribute to the response to environmental factors under natural conditions (Richards et al., 2017). As many developmental pathways are linked to both genetic and epigenetic processes, genetic diversity between populations can be partly linked to the corresponding epigenetic variability (Richards et al., 2012). Knowledge on the mechanisms involved in epigenetic responses may assist in forest genetic resource management and provide opportunities for tree breeding, especially as in long-lived forest trees, notable changes in allele frequencies usually tend to occur rather slowly (Brautigam et al., 2013).

The role of the interaction between genetic and epigenetic diversity in the process of evolution of natural populations, is beginning to be understood (Grativol et al., 2012) and more data are needed (Richards et al., 2017). The present study has attempted to contribute to this field, by studying a black pine population exposed to lignite power plants air pollution for some 70 years. The pivotal finding of this research was that for the first time we showed that in contrast to the absence of differences at the genetic level, statistically significant differences were detected at the epigenetic population level between the most exposed to pollution *P. nigra* population and the control population.

## Data availability statement

The original contributions presented in the study are included in the article/**Supplementary Material**. Further inquiries can be directed to the corresponding author.

## Author contributions

FAA conserved and designed the study. EK has carried out sampling, experimental and data analyses. EA and IG participated in the analyses. FAA, EK and EB evaluated the results. EK and FAA drafted and refined the manuscript. EA, IG and AD, critically read the manuscript. All of the authors have read and approved the manuscript.
