# Supplementary material for: Genetics and epigenetics of Pinus nigra populations with differential exposure to air pollution
Source: Front Plant Sci. 2023 Apr 6;14:1139331. doi: 10.3389/fpls.2023.1139331 (PMC10117940; doi:10.3389/fpls.2023.1139331)
Supplement: Supplementary file 1 [file DataSheet_1.docx]

Genetics and epigenetics of *Pinus nigra* populations with differential exposure to air pollution

Supplementary Material

Table S1. Preselective primers employed in the genetic analyses of *Pinus nigra* populations using f-AFLP markers.

| **Preselective EcoRI** |  |  |  |
| --- | --- | --- | --- |
| **Preselective EcoRI primer+1** | **Unlabeled** | **GACTGCGTACCAATTC-A** | **eth 3** |
| **Preselective MseI primer +1** | **Unlabeled** | GATGAGTCCTGAGTAA-C | eth 10 |

Table S2. Selective primers employed in the genetic analyses of *Pinus nigra* populations using f-AFLP markers.

| **Selective MseI primer +4 No15** | **Unlabeled** | **GATGAGTCCTGAGTAA-CATC** | **Selective EcoRI primer +3** | **ABI-Tamra** | **GACTGCGTACCAATTC-AAG** |
| --- | --- | --- | --- | --- | --- |
|  |  |  | Selective EcoRI primer +3 | ABI-HEX | GACTGCGTACCAATTC-ACT |
| **Selective MseI primer +3 No10** | **Unlabeled** | GATGAGTCCTGAGTAA-CCA | Selective EcoRI primer +3 | ABI-FAM | GAC-TGC-GTA-CCA-ATT-C-ATG |
|  |  |  | Selective EcoRI primer +3 | 5' ROX | GACTGCGTACCAATTC-AAC |

Table S3. Preselective primers employed in the epigenetic analysis of *Pinus nigra* populations using f-MSAP markers.

| **Preselective EcoRI primer+1** | **Unlabeled** | **GACTGCGTACCAATTC-A** | **eth 3** |
| --- | --- | --- | --- |
| **Preselective H,M-T primer** | **Unlabeled** | ATGAGTCTCGATCGG-T | H,M-T |

Table S4. Selective primers employed in the epigenetic analysis of *Pinus nigra* populations using f-MSAP markers.

| **Selective MspI-HpaII primer+3** | **Unlabeled** | **ATGAGTCTCGATCGGTCA** | **eth87** | **Selective EcoRI primer +3** | **ABI-FAM** | **GAC-TGC-GTA-CCA-ATT-C-ATG** | **eth 45** |
| --- | --- | --- | --- | --- | --- | --- | --- |
|  |  |  |  | Selective EcoRI primer +3 | ABI-HEX | GACTGCGTACCAATTC-ACT | eth64 |
|  |  |  |  | Selective EcoRI primer +3 | 5' ROX | GACTGCGTACCAATTC-AAC | eth4b |
|  |  |  |  | Selective EcoRI primer +3 | ABI-Tamra | GACTGCGTACCAATTC-AAG | eth 6b |

Table S5. Average annual precipitation and temperature at the exposed (Mpourika) and control (Ag. Kyriaki) sites of *Pinus nigra* populations in the AKPB.

| **Weather conditions** | **Mpourika*** | **Agia Kyriaki**** |
| --- | --- | --- |
| **Annual precipitation (mm)** | 1017.3 | 1177.3 |
| **Annual mean temperature (**^ο^C) | 13.5 | 12.8 |

*Forest Authority of Veroia (personal communication)

**Katsavouni et al., 2013

Table S6. Results of soil analysis in the exposed (Mpourika) and control (Ag. Kyriaki) sites of the AKPB.

| Soil analysis | | pH | C | Organic matter | Exchangeable cations (cmol/kg) | | | | Extractable micronutrients (μg/g) | | | |
| --- | --- | --- | --- | --- | --- | --- | --- | --- | --- | --- | --- | --- |
|  |  | H_2_O | % | % | Ca | Mg | K | Na | Cu | Fe | Zn | Mn |
| Agia Kyriaki | Mean | 5.48 | 2.18 | 3.76 | 7.31 | 0.66 | 0.20 | 0.23 | 0.19 | 48.27 | 0.63 | 19.22 |
|  | St. dev | 0.37 | 0.80 | 1.38 | 4.66 | 0.30 | 0.07 | 0.05 | 0.08 | 14.45 | 0.38 | 13.53 |
| Mpourika | Mean | 7.66 | 5.08 | 8.71 | 29.37 | 2.23 | 0.60 | 0.62 | 1.13 | 25.31 | 0.76 | 130.59 |
|  | St. dev | 0.05 | 0.62 | 1.14 | 13.85 | 0.02 | 0.09 | 0.12 | 0.07 | 4.72 | 0.21 | 35.07 |

Table S7. Analysis of molecular variance (AMOVA) for AFLP and methylation-sensitive polymorphisms (MSAP) data (all subepiloci) for needles and embryos tissues performed in all populations.

| Loci/groups | Source of variation | d.f. | Variance component | Total variance (%) | Φ-statistics (Φ_PT_) | *p* value |
| --- | --- | --- | --- | --- | --- | --- |
| AFLP loci | Among populations | 1 | 18,208 | 0% | **0.034** | 0.001 |
|  | Within populations | 190 | 3104,750 | 100% |  |  |
|  | Total | 191 | 3122,958 | 100% |  |  |
| MSAP *n-*subepiloci (needles) | Among populations | 1 | 49,283 | 3% |  |  |
|  | Within populations | 46 | 1228.925 | 97% |  |  |
|  | Total | 47 | 1278.208 | 100% |  |  |
| MSAP *h*-subepiloci (needles) | Among populations | 1 | 17.606 | 3% | 0.027 | 0.002 |
|  | Within populations | 46 | 482.915 | 97% |  |  |
|  | Total | 47 | 500.521 | 100% |  |  |
| MSAP *m-*subepiloci (needles) | Among populations | 1 | 30.254 | 4% | 0.041 | 0.001 |
|  | Within populations | 46 | 691.475 | 96% |  |  |
|  | Total | 47 | 721.729 | 100% |  |  |
| MSAP *all*-subepiloci (needles) | Among populations | 1 | 49,283 | 3% | **0.034** | 0,001 |
|  | Within populations | 46 | 1228,925 | 97% |  |  |
|  | Total | 47 | 1278,208 | 100% |  |  |
| MSAP *n*-subepiloci (embryos) | Among populations | 1 | 17.479 | 4% |  |  |
|  | Within populations | 46 | 424.625 | 96% |  |  |
|  | Total | 47 | 442.104 | 100% |  |  |
| MSAP *h*-subepiloci (embryos) | Among populations | 1 | 17.479 | 4% | 0.036 | 0.001 |
|  | Within populations | 46 | 424.625 | 96% |  |  |
|  | Total | 47 | 442.104 | 100% |  |  |
| MSAP *m-*subepiloci (embryos) | Among populations | 1 | 16.521 | 1% | 0.011 | 0.044 |
|  | Within populations | 46 | 599.375 | 99% |  |  |
|  | Total | 47 | 615.896 | 100% |  |  |
| MSAP *all*-subepiloci (embryos) | Among populations | 1 | 35,854 | 2% | **0.021** | 0,001 |
|  | Within populations | 46 | 1086,708 | 98% |  |  |
|  | Total | 47 | 1122,563 | 100% |  |  |

Table S8. Comparison (t-tests) of epigenetic diversity (Shannon index, I_epi_), between the exposed population (Mpourika) and the control population (Ag. Kyriaki) for needle and embryo samples.

| **Population** | **Needles** | **Embryos** |
| --- | --- | --- |
| **Mpourika** |  |  |
| I_epi_ | 0.090 | 0.118 |
| **SE** | 0.004 | 0.005 |
| **Agia Kyriaki** |  |  |
| I_epi_ | 0.107 | 0.102 |
| **SE** | 0.004 | 0.005 |
| **t test** | 2.999 | 2.2587 |
| **p** | 0.0043 | 0.0287 |

Table S9. Comparison of the Shannon diversity index values for genetic (AFLP) and epigenetic (MSAP) variation between the exposed (Mpourika) and control (Ag. Kyriaki) populations.

| **Needles** | **AFLP** | **MSAP** |
| --- | --- | --- |
| **Population** | I | I_epi_ |
| **Mpourika** | 0.123 | 0.090 |
| **SE** | 0.08 | 0.04 |
| **Agia Kyriaki** | 0.134 | 0.107 |
| **SE** | 0.08 | 0.004 |
| **Mean** | 0.129 | 0.099 |
| **SE** | 0.03 | 0.03 |
